# High altitude hypoxia as a factor that promotes tibial growth plate development in broiler chickens

**DOI:** 10.1371/journal.pone.0173698

**Published:** 2017-03-10

**Authors:** Shucheng Huang, Lihong Zhang, Mujeeb Ur Rehman, Muhammad Kashif Iqbal, Yanfang Lan, Khalid Mehmood, Hui Zhang, Gang Qiu, Fazul Nabi, Wangyuan Yao, Meng Wang, Jiakui Li

**Affiliations:** 1 College of Veterinary Medicine, Huazhong Agricultural University, Wuhan, People's Republic of China; 2 Laboratory of Detection and Monitoring of Highland Animal Disease, Tibet Agriculture and Animal Husbandry College, Linzhi Tibet, People's Republic of China; 3 Faculty of Veterinary & Animal Sciences, Lasbela University of Agriculture, Water and Marine Sciences Uthal, Balochistan, Pakistan; Sichuan University, CHINA

## Abstract

Tibial dyschondroplasia (TD) is one of the most common problems in the poultry industry and leads to lameness by affecting the proximal growth plate of the tibia. However, due to the unique environmental and geographical conditions of Tibet, no case of TD has been reported in Tibetan chickens (TBCs). The present study was designed to investigate the effect of high altitude hypoxia on blood parameters and tibial growth plate development in chickens using the complete blood count, morphology, and histological examination. The results of this study showed an undesirable impact on the overall performance, body weight, and mortality of Arbor Acres chickens (AACs) exposed to a high altitude hypoxic environment. However, AACs raised under hypoxic conditions showed an elevated number of red blood cells (RBCs) and an increase in hemoglobin and hematocrit values on day 14 compared to the hypobaric normoxia group. Notably, the morphology and histology analyses showed that the size of tibial growth plates in AACs was enlarged and that the blood vessel density was also higher after exposure to the hypoxic environment for 14 days, while no such change was observed in TBCs. Altogether, our results revealed that the hypoxic environment has a potentially new role in increasing the blood vessel density of proximal tibial growth plates to strengthen and enhance the size of the growth plates, which may provide new insights for the therapeutic manipulation of hypoxia in poultry TD.

## Introduction

The Tibetan plateau is a high altitude geographical region of China with an average elevation of more than 4,000 meters. This region is generally known for its extreme environmental conditions, including low oxygen content, low barometric pressure, and great temperature fluctuations on a daily basis. These conditions impose severe physiological challenges on endothermic animals [1,2]. The typical stress at high altitudes is hypoxia, which is caused by the fall in barometric pressure with increasing altitude and consequently lower oxygen content in the inspired air compared to sea level [1]. Furthermore, due to the slow growth rate of Tibetan chickens (TBCs), it has been a common practice for most poultry farmers in Tibet to raise commercial broiler chickens (such as Arbor Acres chickens) because of their fast growth rate. However, these chickens are not well adapted to the hypoxic environment, which is the main ecological factor with a negative impact on the animal’s health and a threat to their survival at high altitudes [3].

Hypoxia refers to low partial pressure of oxygen (O_2_) in the inspired air and threatens the survival, development, and reproduction of both humans and animals [3–10] because of its physiological challenges on the body. Jia *et al*. [10] have revealed the unique physiological responses and adaptation mechanism of animals in response to high altitude hypoxia. Chickens provide a proper model to study physiological adaptations under hypoxic stress conditions, and TBCs are a unique aboriginal breed that has undergone selection for such trials to inhabit the high altitude Tibetan plateau. TBCs are one of the native poultry breeds that have been found on the Tibetan plateau (2,600 m~4,500 m above sea level) for approximately 1000 years. Therefore, this breed has the ability to adapt to rigorous environmental conditions such as low air pressure and partial pressure of oxygen [11–13].

In comparison to the chicken breeds at lower altitudes, TBCs have the adaptability to surmount the extremely harsh environments due to their elevated number of red blood cells and blood hemoglobin level [11]. Most importantly, this unique breed has never been reported to have any leg disorders, especially Tibial dyschondroplasia (TD). There is a high incidence rate of TD in meat-type and fast-growing poultry, especially turkeys, with up to 80% developing TD at the age of 12 weeks [14]. Numerous studies have reported that TD is a bone abnormality. The lesions of TD are characterized by the presence of an irregular, white, opaque, unmineralized and unvascularized mass of cartilage that is attributable to cell death, no blood supply and degenerative changes in the proximal end of the tibia and that is the leading cause of osteomyelitis, osteochondrosis, and lameness in poultry [14–19]. Altogether, these factors lead to significant economic losses to the poultry industry and compromise poultry welfare. In addition, it has been reported that normal avian growth plates consist of long columns of chondrocytes that are well vascularized with more cellular zones compared to mammalian growth plates [19–21]. Moreover, the growth plate regulates bone ossification and elongation by maintaining the balance between chondrocyte proliferation and differentiation [22,23]. However, the etiology of TD linked to the development of the growth plate is still unknown. Rath *et al*. [16] proposed that the possible pathogenesis of TD is linked to abnormal cell death (apoptosis) in the growth plates, and these dead chondrocytes cannot be removed promptly due to sparse vascularity of the growth plate.

Numerous studies have highlighted the role of hypoxia in initiating the expression of hypoxia-induced factor-1 (HIF-1α) and further inducing the expression of target genes such as vascular endothelial growth factor (VEGF) and its receptors, which promote various systemic physiological changes including angiogenesis and vascular development [24–29]. Angiogenesis plays a crucial role in the homeostatic mechanisms associated with the vascular oxygen supply in hypoxia. However, the mechanism of hypoxia-induced tibial growth plate development and function remains unclear. Therefore, this study was designed to understand the physiological mechanism of hypoxia in the development of the growth plate and to understand the possible association of angiogenesis and vascular development mechanisms with high altitude hypoxia during the early stages of broiler growth in AACs and TBCs, which may provide new insights for the therapeutic manipulation of hypoxia to prevent lameness in these birds.

## Materials and methods

### Ethics statement

All the experiments were approved and reviewed by the Animal Welfare and Ethics Committee of the Huazhong Agricultural University Wuhan, China (approval permit number: 31272517). The animal experiments and procedures were performed in strict accordance with the relevant guidelines of PSCH (No. 5 Proclamation of the Standing Committee of Hubei People’s Congress, P.R. China). Notably, none of the chickens exhibited signs of illness or distress prior to their death. However, to minimize suffering, the chickens were euthanized using pelltobarbitalum injections with standard protocols if they exhibited specific signs of illness during the experiment.

### Chicken husbandry

One-day-old healthy AACs (n = 120) were purchased from a commercial hatchery of Chengdu, China (average altitude, 500 m), and transported to a laboratory of the Tibet Agricultural and Animal Husbandry College (average altitude, 2,986 m above sea level) on the same day. Simultaneously, one-day-old healthy TBCs (n = 120) were also purchased from a commercial hatchery at Lhasa (average altitude, 3,651 m) and transported to the same laboratory. All the chicks were randomly allocated into two groups by birth weight, namely, the hypobaric normoxia group and the hypoxia group (approximately 21% oxygen content and natural oxygen content, respectively; n = 60/group, 4 cages per treatment and 15 chicks per cage). The oxygen content of the hypobaric normoxia group was maintained with an oxygenator (Yuwell, Suzhou, China). Moreover, the oxygen content of the normoxia and hypoxia groups was monitored with a gas detector (CY-7B, Oxygen analysis instrument factory, Jiande, China) throughout the experiment.

**Fig 1:**
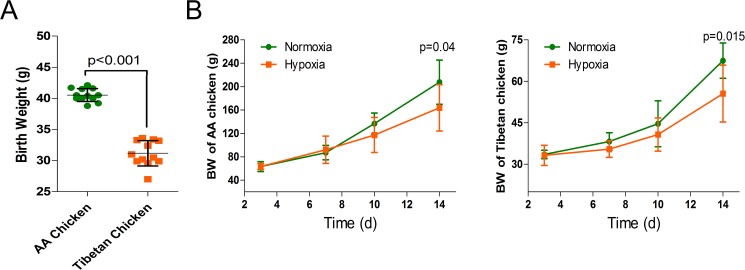
Effects of high altitude hypoxia on the birth weight and body weight of AACs and TBCs. (A) Effect of high altitude hypoxia on the birth weight of AACs and TBCs (n = 12). (B) Effect of high altitude hypoxia on the body weight of AACs and TBCs (n = 8). The data are expressed as the mean±SD. *p<0.05, normoxia group vs. hypoxia group. BW, body weight.

All the AACs (40.5±1.02 g) and TBCs (31.2±2.03 g) had similar initial weights or birth weights (Fig 1A), and the nutrient contents of the diets (12.6 MJ metabolizable energy/kg of diet, 220 g/kg crude proteins) were maintained as suggested by the National Research Council (NRC, 1994). The nutrient composition of the broiler diet is shown in S1 Table. The chicks were raised in two-layer metal cages (size, 80 cm×60 cm×50 cm) for 14 days. Four times a day (every 6 h), their diet, drinking water, and overall performance were monitored; lack of clustering and no difficulty in breathing were considered normal performance for the chickens. The brooding temperature was maintained between 33°C and 35°C during the first week and gradually decreased up to 29°C by the end of second week. The daily light/dark cycle was fixed at 23 h light and 1 h dark during the whole experiment. In addition, feed and water were provided *ad libitum*.

### Production performance analysis

The chicks were group-weighed on day 3, day 7, day 10, and day 14 with the cages, and the average daily weight gain (ADG) and average daily feed intake (ADFI) were calculated per group. Feed consumption (FC) was also determined on day 3, day 7, day 10, and day 14 with the cages, and feed consumption per chick (g/chick) was calculated by dividing the total FC of each cage by the actual number of chicks in that cage. The feed conversion ratio (FCR) was determined as the FC per body weight gain (g/g) per cage per time. Mortality (no breathing, no heartbeat) was recorded on a daily basis.

### Blood parameters

Before euthanasia, blood samples were obtained through wing veins using heparinized syringes. All groups were analyzed for red blood cell (RBC) count, hemoglobin (Hb) level and hematocrit (Hct) values. These parameters were determined using an automatic blood analyzer (XFA6000, Pulang Company, Nanjing, China) that was standardized for the analysis of chicken blood parameters.

### Morphology and histology of the tibial growth plates

Two birds per treatment cage (n = 8/treatment) were randomly selected on day 3, day 7, day 10, and day 14 of the experiment. The stripping of the tibial longitudinal muscles and preparation of sagittal sections of the proximal tibial growth plates were performed to analyze the morphology as previously described by Rath *et al*. [30]. The collected tibial bone samples were fixed in 4% paraformaldehyde at 4°C in PBS and decalcified in 10% EDTA. After the samples were dehydrated in ethanol and cleared in xylene, all the samples were embedded in paraffin, and histological sections of 4~5 μm thickness were prepared and stained with hematoxylin and eosin for microscopic examination as previously described [19,31].

### Statistical analysis

Statistical analyses of the data were performed using SPSS Statistics Version 17.0 software for windows (SPSS Inc., Chicago, IL). Comparisons between two groups were performed using one-way ANOVA followed by Duncan’s test. For mortality, a χ^2^ analysis was performed for each group. Differences were considered statistically significant at *p*<0.05, and the values were presented as the means±SD or SEM.

## Results

### Overall performance of the chickens

There was no significant difference in the body weight (BW) of AACs and TBCs per treatment group from day 1 to day 10 of the experiment. However, the BW of AACs and TBCs raised under hypoxic conditions were significantly lower on day 14 (*p* = 0.04 and *p* = 0.015, respectively) compared to the normoxia group. Furthermore, the effects of hypoxia on the broilers were progressively severe (Fig 1B).

**Fig 2:**
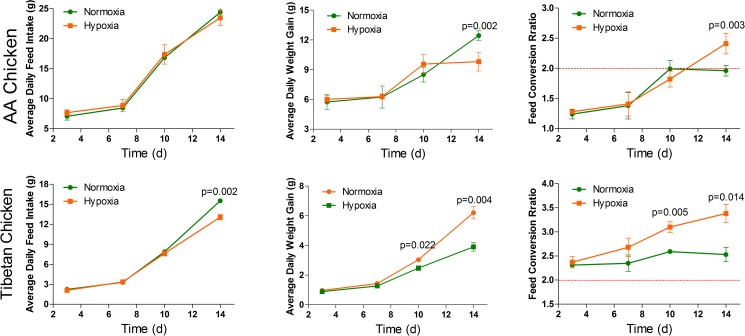
Effect of high altitude hypoxia on the overall performance of AACs and TBCs (n = 4 cages). The value for the red line as the reference in this figure is 2. The data are expressed as the mean±SD. *p<0.05, normoxia group vs. hypoxia group.

As shown in Fig 2, there was no significant difference in the average daily feed intake (ADFI) of the AACs compared to the normoxia group during the 14 days of experiment. Similarly, there were no significant changes in the average daily weight gain (ADG) and feed conversion ratio (FCR) of the AACs, except on day 14 (*p* = 0.002 and *p* = 0.003, respectively) between the normoxia group and hypoxia group. However, the ADFI of the TBCs was significantly decreased (*p* = 0.002) during the last four days (day 10~14) of the experiment. In contrast, ADG was significantly decreased in the TBCs of the normoxia group and hypoxia group (*p* = 0.022 and *p* = 0.004, respectively). Conversely, FCR was significantly increased in the TBC normoxia group and hypoxia group on day 10 and day 14 (*p* = 0.005 and *p* = 0.014, respectively).

### Chicken mortality rate

**Fig 3:**
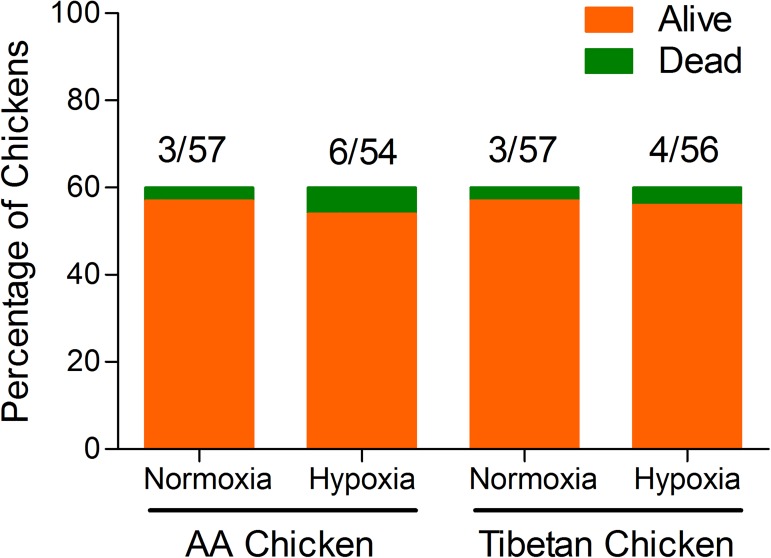
Effect of high altitude hypoxia on the mortality rate of AACs and TBCs. χ^2^ analysis was performed on the number of chickens who died throughout the experiment.

The rate of mortality in the AACs and TBCs was 7.5% (9/120) and 5.83% (7/120), respectively, throughout the experiment (1~14 days). The mortality per treatment group (normoxia group and hypoxia group) is illustrated in Fig 3. Although no significant difference was observed in each treatment group of the AACs and TBCs (*p* = 0.30, OR = 2.11; *p* = 0.70, OR = 1.36, respectively), the rate of mortality was higher for AACs than TBCs under the same conditions.

### Blood parameters

**Fig 4:**
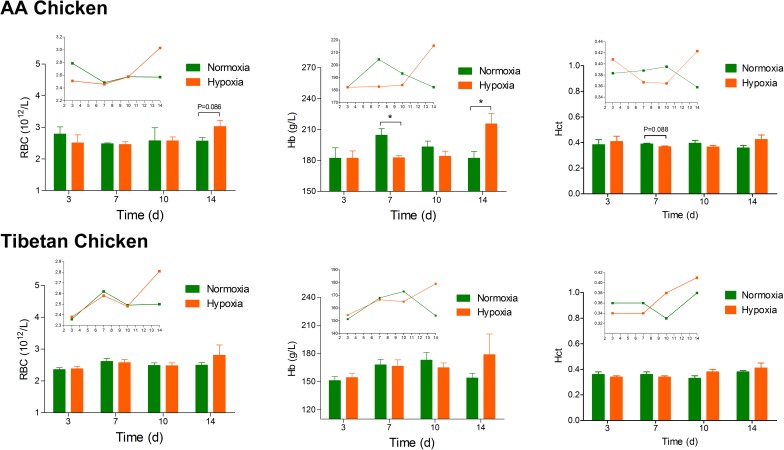
Effect of high altitude hypoxia on the blood parameters of AACs and TBCs (n = 4). The data are expressed as the mean±SEM. *p<0.05, normoxia group vs. hypoxia group. RBCs, red blood cells; Hb, hemoglobin; Hct, hematocrit.

In this study, there was no significant difference between the normoxia group and hypoxia group. However, a rising tendency in the total RBC count, Hb level and Hct values (except Hb levels of AACs) was observed among all the AAC and TBC groups from day 10. The Hb level of AACs in the hypoxia group was significantly different on day 7 and day 14 (*p* = 0.024 and *p* = 0.033, respectively) in comparison to that of the normoxia group. In contrast, hypoxia had a more apparent impact on the blood parameters (RBC and Hb) of AACs compared to those of TBCs (Fig 4).

### Morphological changes in the tibial growth plates

**Fig 5:**
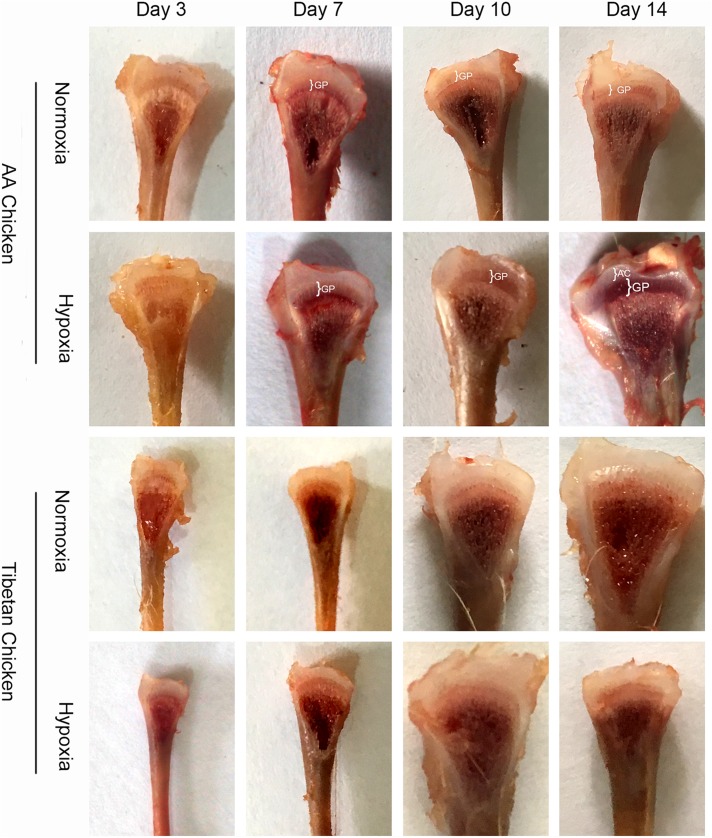
Effect of high altitude hypoxia on the morphology of the growth plates in AACs and TBCs. The enlarged growth plates in the hypoxia group were compared with normoxia group. AC, articular cartilage; GP, growth plate.

To examine the development of proximal tibial growth plates, eight chickens were randomly selected and sacrificed from each group (normoxia group and hypoxia group) on day 3, day 7, day 10, and day 14. As shown in Fig 5, the widths of the proximal tibial growth plates of AACs were markedly enlarged on day 10 and day 14 in the hypoxia group. However, the widths of the tibial growth plates of TBCs were not enlarged compared to the normoxia group. In addition, the tibial growth of AACs was much faster compared to TBCs.

### Histological examination of the tibial growth plates

**Fig 6:**
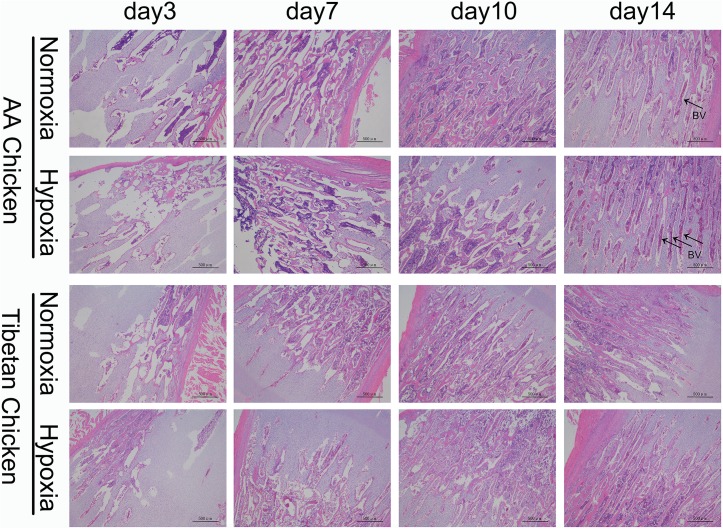
Histological examinations of the growth plates in both AACs and TBCs at high altitude. Obvious increase in the density of the metaphyseal blood vessels on day 14 in the hypoxia group compared to the normoxia group. The arrows indicate blood vessels. BV, blood vessels. Scale bar = 500 μm.

Histological analysis of the proximal tibia of AACs showed a significant increase in the density of metaphyseal blood vessels on day 14 in the hypoxia group compared to the normoxia group. However, no obvious changes in TBCs were observed (Fig 6).

## Discussion

TBCs are an aboriginal breed with a history of thousands of years of living in high altitude areas and are characterized by their small size and low birth weight, which may be attributed to their better adaptation to altitudes of more than 4,000 m (Fig 1A). The findings of the present study indicated the effect of high altitude hypoxia on suppressing the BW of AACs and TBCs in comparison with the normoxia group. These observations confirmed the findings of Gao *et al*. [32], which indicated reduced body weight gain during hypoxia. This reduced body weight gain might be due to a reduction in the nutritional energy intake or intestinal energy uptake as a result of impaired intestinal function and increased energy expenditure [3]. Hypoxia directly leads to systemic hypoxemia and an imbalance between the animal’s demand for O_2_ and the insufficient O_2_ available, resulting in a decrease in BW at high altitudes. Semenza [33] reported that an inadequate supply of O_2_ affects both physiological performance and growth capacity. Altogether, our findings also demonstrated the various effects of high altitude hypoxia on the overall performance of TBCs and especially AACs.

The final BW values of AACs and TBCs in the hypoxia group at 2,986 m above sea level on day 14 were 163.75 g and 55.54 g, respectively, similar to the findings of Li *et al*. [3], where the BW of AACs raised at high altitude was approximately 172.6 g on day 14. Meanwhile, previous reports on the BW of TBCs had lower numbers, but the ADFI remained the same as the AACs. Similarly, Westerterp *et al*. [34] suggested that energy intake is the dominant determinant of body weight loss for humans under hypoxic conditions at high altitudes. Similar observations were made by De Grauw *et al*. [35] and Camm *et al*. [36], who demonstrated that exposure to hypoxia can lead to a significant decrease in food intake. The reduction in food intake may not solely account for the decrease in BW, as Li *et al*. [3] indicated that the villi height and crypt depth of AACs reared at high altitude were also reduced compared to those reared at low altitudes. Altogether, these findings suggest that the absorption of nutrients at high altitudes could be compromised; thus, hypoxia affects not only ADFI but also ADG. In addition, a surprising and inspiring finding from this study was the value of FCR, which was lower than 2 between days 3~10 and markedly increased on day 14 under hypoxia conditions, suggesting the induction of feed conversion to body mass. This observation is likely in agreement with the report of Li *et al*. [3], who found that the FCR of broilers reared at high altitudes was 2.21 on day 14. Nevertheless, the FCR was also less in the TBCs. In this study, we noticed that the FCR of TBCs was higher than that of AACs during all four readings and that the value of FCR was greater than 2, even up to 3.5, suggesting the prolong growth period of TBCs and low economic benefits caused by the hypoxic environment.

The present experimental study on the production performance of broilers showed that hypoxia not only affects the birth weight, BW, ADFI, ADG, and FCR but also slightly affects the survival ratio of both AACs and TBCs. Similar observations were made by Visschedijk [37], who studied lowland chickens raised at high altitudes where inadequate O_2_ exchange resulted in hypoxic syndrome. In general, proper ventilation is an important strategy to avoid high death rates in lowland chickens raised at high altitudes [38]. Noticeably, hypoxia affects the survival rates of both embryos and lowland chickens at high altitudes. The results of the present study did not indicate any significant difference in the mortality rates of AACs and TBCs exposed to hypoxia or normoxia during the rearing period. However, hypoxia is known as a major risk factor for the death of broilers, especially AACs.

Blood can be affected by hypoxia acts as transports oxygen to the organs of the body, and its parameters are very critical in evaluating animal physiology under hypoxic conditions [39–41]. In particular, Hb acts as a hypoxic sensor, along with the RBCs, to perform the fundamental physiological process of O_2_ delivery to the hypoxic tissues [39,42]. Another study by Liu *et al*. [43] showed that the Hb concentration is the most important factor responsible for ensuring oxygen concentration in the blood under hypoxic conditions. In the present study, we found that the hypoxic environment significantly increased the Hb levels on day 14 compared to the normoxia group in AACs, and similar changes in Hb have also been observed in avian embryos [44]. Furthermore, an increasing tendency in the RBC number, Hb level and Hct values was observed at days 10~14 in both the AAC and TBC groups during the entire experiment. These effects could be due to an increase in the oxygen demand of the birds for respiration and normal physiological processes, and the increased RBC number, Hb concentration, and Hct volume can be attributed to the oxygen compensatory effects. However, hypoxia had a more pronounced impact on the blood parameters of AACs compared to TBCs, suggesting that the TBCs have good adaptability to hypoxic conditions. In contrast, hypoxia influenced the bone marrow of the broiler chickens to increase the RBC number and Hb concentration and to increase the blood vessel number in the face of imminent hypoxia. Therefore, further studies are required to confirm the effects of hypoxia on the growth and development of tibial growth plates in relation to the numbers of blood vessels [45].

The most recommended methods for the assessment of tibial growth plate development and pathology are TUNEL assays and hematoxylin and eosin staining [19,31,46,47]. Morphological examination of the tibial anatomy includes envisioning the width of the tibial growth plate, which is considered an alternative method for the assessment of tibia development [19,30]. Additionally, focusing on the production performance of the birds, all parameters, including mortality rates, of both the AACs and TBCs at high altitudes were found to be closely related to hypoxia. These findings suggest that hypoxia has a negative effect on the growth of broiler chickens at high altitudes. However, the hypoxia group, especially the AACs that were constantly kept under hypoxic conditions for 14 days, showed a surprising increase in the tibial growth plate size (morphologically). At the same time, the histology of the tibial growth plates on day 14 showed higher blood vessel densities. Previous studies have indicated that the disturbance of blood vessels on the growth plate decreases bone mineralization and hypertrophic chondrocyte replacement [48,49]. Moreover, Lee *et al*.[50] indicated that the increased parameters of bone formations were closely related to high blood vessel number and density. Similar results were reported by Zhao *et al*. [51], who highlighted the role of hypoxia in inducing chondrogenesis and angiogenesis, as well as its role in the bone repair process. Altogether, these findings suggest that bone formation is largely dependent on vascularization. Thus, hypoxia-induced angiogenesis for the formation of tibial growth plates is of great importance. However, in this study we found that the width of tibial growth plates and the density of the metaphyseal blood vessels in the proximal tibia of TBCs did not show any apparent change, unlike AACs, which may be attributable to the adaptability of the TBCs to long-term hypoxic conditions and a possible reason for the nonexistence of TD in these birds.

Unlike human beings, hypoxia-induced weight loss in chickens [34,52,53] is beneficial in terms of their fitness. However, the external hypoxic environment may also prevent the occurrence of TD. Nevertheless, to pursue the largest economic benefits in large-scale poultry breeding, it is important that the birds gain more BW; therefore, possible drug applications are required to obtain hypoxic conditions [51]. Moreover, studies by Hsieh *et al*. [54] reported that such drugs can prolong the activity of HIF-1α in the bloodstream and lead to an increase in the endogenous production of erythropoietin, which may enhance chondrogenesis and vascular formation.

In summary, our results showed the extensive effects of hypoxia at high altitudes on the overall performance of poultry and on the development of the tibial growth plates in particular. We proposed that hypoxia not only has a negative effect of the growth performance of broilers but also plays an important role in the enlargement of tibial growth plate sizes and in the increase in metaphyseal blood vessel density to the proximal tibia. Altogether, these findings may provide new insights for the therapeutic manipulation of hypoxia in poultry TD and have important implications for the pathophysiology of tibial growth plates under hypoxic conditions. However, the expression of related genes responsible for the increase in the width of the growth plates and for the corresponding vascular density is unclear. Further studies on the possible functional effects of growth plate-related genes, including HIF-1α, VEGF, and its receptors (such as VEGFR1 and VEGFR2), are required.

## Supporting information

S1 Table Nutrient composition of the broiler diet. (DOC)
