# Supplementary material for: High altitude hypoxia as a factor that promotes tibial growth plate development in broiler chickens
Source: PLoS One. 2017 Mar 10;12(3):e0173698. doi: 10.1371/journal.pone.0173698 (PMC5345845; doi:10.1371/journal.pone.0173698)
Supplement: S1 Table — (DOC) [file pone.0173698.s001.doc]

| Item | Content |
| --- | --- |
| Crude protein, ≥% | 21.0 |
| Crude fibre, ≤% | 5.0 |
| Ash, ≤% | 7.0 |
| Calcium, % | 0.80~1.30 |
| Phosphorus, ≥% | 0.50 |
| NaCl, % | 0.30~0.80 |
| Water, ≤% | 14.0 |
| Methionine, ≥% | 0.48 |

Table S1 Nutrient composition of broiler diets
